# Preparation, Characterization, and In Vitro Bioactivity of *Artemisia capillaris* Thunb. Leaf Protein Peptide Selenium Chelates

**DOI:** 10.1155/ijfo/4720991

**Published:** 2025-12-17

**Authors:** Wen-Lu Wei, Wen-Jun Wang, Chuan-Long Yu, Hui Chen, Su-Yun Lin, Ling-Li Chen

**Affiliations:** ^1^ Jiangxi Provincial Key Laboratory of Natural Products and Functional Foods, College of Food Science and Engineering, Jiangxi Agricultural University, Nanchang, Jiangxi, China, jxau.edu.cn

**Keywords:** antioxidant activity, *Artemisia capillaris* Thunb, cytotoxicity, peptide selenium chelate, structural identification

## Abstract

This study aimed to prepare *Artemisia capillaris* Thunb. leaf protein peptide selenium chelates (AP‐Se) employing sodium selenite as a selenium (Se) source, with a focus on their structure and biological activities. *Artemisia capillaris* Thunb. leaf protein (AP) was extracted from *Artemisia capillaris* Thunb. leaf (AC) and enzymatically digested to obtain AP peptide (AP‐P). The results exhibited that the maximum Se chelating capacity of 52.78 ± 0.74 (mg/g) was obtained at a peptide‐Se mass ratio of 1:3.3, pH 9, and temperature of 49°C condition. The N‐terminal amino group and C‐terminal carboxyl group of AP‐P were the main binding sites of selenium ions. The chelation reaction caused folding and curling of the peptide chain in AP‐P, which produced folds and crystal in AP‐Se microstructure. Furthermore, at certain dosages, AP‐Se demonstrated an ABTS^+^ clearance rate comparable with that of Vitamin C (VC), along with outstanding gastrointestinal permeability. Furthermore, AP‐Se was proven to be less hazardous to cells than sodium selenite. Overall, the study indicated that AP‐Se may have a promising usage as an effective natural selenium supplement in the medical and food industries.

## 1. Introduction

Selenium (Se) is considered as one of the most essential trace elements for growth and maintenance of immunological function in both humans and animals, which plays a pivotal role as a redox signal in influencing oxidative stress, inflammation, and lipid metabolism [1]. The World Health Organization (WHO) recommends a Se intake of 30~50 *μ*g/d [2]. However, approximately a billion people worldwide are deficient in Se [3]. Se deficiency is commonly closely associated with human diseases such as cancer, Keshan disease, Kaschin–Beck disease, immune responses, and abnormal thyroid hormone function [4].

At present, the selenium supplement products on the market are mainly divided into inorganic selenium and organic selenium. Compared with inorganic Se, organic Se, because of its high safety, better bioactivity, and bioavailability, has attracted extensive attention from national and international scholars [5]. The biofortification of Se is the biological conversion of inorganic Se to organic Se through living organisms (plants, animals, microorganisms), but the conversion period was long and the conversion rate was uncontrollable, may also result in environmental contamination and resource waste [6]. Artificial organic Se is produced by chelating plant or animal hydrolyzed peptides with sodium selenite. Compared with biofortified Se, artificial organic Se is environmentally friendly, with high conversion rate, low cost, and excellent bioavailability. Hence, artificial organic Se has become hotspot in the field of nutritional research in recent years [7].

Protein peptides from different sources, such as bovine bone collagen peptides, Europen eel (*Anguilla anguilla*) bone peptide, cationic peptides, and fish collagen peptide, chelated with mineral elements (Mg^+^, Ca^2+^, Cu^2+^, Ni^2+^, and Zn^2+^) not only improve the bioavailability of the mineral elements, but also give the chelated compounds better bioactivities, such as antioxidant [5], antitumour [7], anti‐inflammatory [8], and antimicrobial [9], etc. However, peptide selenium chelates were understudied compared with Mg^+^, Ca^2+^ and Zn^2+^, etc. Ye et al. [5] revealed that the soybean isolated peptide (SPIP)‐Se chelates (SPIP‐Se) have better antioxidant activities in vitro than SPIP, which could also repair oxidative damage in Caco‐2 cells by increasing antioxidant enzyme activities. Furthermore, Xiong et al. [7] discovered that the peptide‐Se chelate formed by chelating the specific peptide (RLA) in *Cardamine violifolia* protein hydrolysate with Se could positively regulate various biological processes in human Coca‐2 cells, as well as exert specific anticancer effects by modulating apoptosis. Therefore, the investigation of peptide selenium chelates is significant for the development of new low‐toxicity and efficient selenium supplements.

As one of the most significant edible herbs in China, *Artemisia capillaris* Thunb. (AC) is widely distributed in Asia and Europe, which was first mentioned in the Divine Farmer’s *materia medica* [10, 11]. AC contains a variety of bioactive activities, including hepatoprotective and cholinergic [12, 13], anticancer, and anti‐inflammatory [14, 15]. Our preliminary research indicated that AC contained more protein and less fat, and the amino acid profile of AC and its leaf protein extracts meets [16] protein quality standards, which suggested that AC has the potential to be a sustainable alternative protein source [17]. Our previous study also revealed that AP (*Artemisia capillaris* Thunb. leaf protein) possesses better antioxidant capacity, and the antioxidant capacity is stronger after hydrolysis [17]. However, there have been no reported AP studies on peptide selenium chelates.

Thus, the objective of this study was to create AP‐Se (AP‐P selenium chelates) utilizing AP‐P (*Artemisia capillaris* Thunb. leaf protein peptide) as a resource and characterize its structure. The biological activities of AP‐Se such as antioxidant activity, digestive properties, and cytotoxicity were investigated. The bioactivities were also compared with those of sodium selenite. This study will provide a theoretical foundation for the development of novel organic Se dietary supplements and the high value utilization of AP.

## 2. Materials and Methods

### 2.1. Reagents

Cell Counting Kit‐8 (CCK‐8) and fetal bovine serum (FBS) were purchased from Beyotime Biotechnology (Shanghai, China). Dulbecco’s Modified Eagle Medium (DMEM) was purchased from Thermo Fisher Scientific (MA, United States). Bile salts, penicillin–streptomycin solution, and sodium selenite were purchased from Solarbio. (Beijing, China). Salivary amylase (12 U/mg), pepsin (3 U/mg), and Trypsin (250 U/mg) in pancreatin were obtained from Yuan Ye Biotechnology Corporation (Shanghai, China). Potassium bromide and 3,3′‐diaminobenzidine were purchased from Macklin Biochemicals Co. (Shanghai, China). NaOH, HCl, ethylene diamine tetraacetic acid (EDTA), KCl, KH_2_PO_4_, NaHCO_3_, NaCl, MgCl_2_, (NH_4_)_2_CO_3_, and CaCl_2_ were purchased from Xilong Science Co. (Guangdong, China). Human hepatocellular carcinomas cells (HepG2), mouse microglia cells (BV2), and human embryonic kidney 293T cells (HEK 293T) were obtained from Jiangxi Agricultural University, and passed for 20~40 generations. All other chemicals were analytical grade.

### 2.2. AP‐P Preparation

AP was prepared according to our previous method [17]. The AP‐P manufacturing process was based on Tang et al.’s method, with modifications [4]. Briefly, 2.5% AP solution was adjusted to pH 8, and 3% (w/v) alkaline protease was added. Enzymatic hydrolysis was done at 55°C for 5 h and inactivated at 100°C for 5 min. The mixed solution was centrifuged at 4000 g for 20 min (4°C) and the supernatant was collected and lyophilized to yield AP‐P.

### 2.3. Optimization of Preparation Conditions for Peptide‐Se Chelates

In terms of parameters that influence the progression of a chemical reaction, five major factors were chosen to optimize the preparation condition of AP‐Se: peptide concentration, peptide‐Se mass ratio, chelating temperature, chelating pH, and chelating duration. Single‐factor tests were carried out with one component changing at different levels while the other factors remained constant. Based on the single factor test, three factors of peptide‐Se mass ratio (X_1_), chelation pH (X_2_), and chelation temperature (X_3_) were selected to optimize by Box–Behnken design (BBD), with Se chelating capacity (Y) as the response value. The parameter levels were shown in Table 1. The AP‐Se was made by the optimized conditions and freeze‐dried for all the following experiments.

**Table 1:** Coded values and corresponding actual values of the variables used in Box–Behnken design.

**Value**	**−1**	**0**	**1**
X_1_	1:2	1:3	1:4
X_2_	8	9	10
X_3_	40	50	60

*Note:* X_1_: peptide‐selenium mass ratio, X_2_: pH, X_3_: temperature.

### 2.4. Se Chelating Capacity Assay

Se content was measured by 3,3′‐diaminobenzidine colorimetry [5]. The concentration of sample was calculated by the Se standard curve equation y = 0.0501x + 0.0137 (*R* ^2^ = 0.9996). The Se chelating capacity was calculated by the following formula:

Se chelating capacitymg/g=ρ/mN×1000

where *ρ* is the Se concentration (*μ*g/mL) in the digested AP‐Se solution; *m* is the weight of the sample (g), *N* is the ratio of sample volume (1 mL) to the volume after dilution (50 mL), where *N* = 0.02 in this study.

### 2.5. AP‐Se Structural Characterization

#### 2.5.1. Ultraviolet (UV) Spectroscopy

The UV spectra in the range of 240~320 nm for AP‐P and AP‐Se solutions (0.125 mg/mL) were acquired by a UV spectrophotometer (Metash, China).

#### 2.5.2. Fourier‐Transform Infrared (FTIR) Spectroscopy

The dried 1 mg AP‐P or AP‐Se were mixed with KBr (100 mg), milled into a powder, then put into a mold and pressed into a transparent sheet, which was scanned by an infrared spectrometer in the range of 500~4000 cm^−1^ (Thermo Fisher Scientific, United States).

#### 2.5.3. Circular Dichroism (CD) Measurements

AP‐P and AP‐Se solutions (1 mg/mL) were prepared and scanned at 1 nm/s over the 190~250 nm wavelength range with a path length of 0.1 cm. The secondary structure and data were analyzed with the BeStSel (Beta Structure Selection) software.

#### 2.5.4. X‐Ray Diffraction (XRD) Analysis

The XRD of AP‐P and AP‐Se were examined by XRD (BRUKER, German) with a scanning speed of 4° min^−1^ ranging from 5 to 75°, stepwise increment of 1°, and an emission slit of 0.3 mm.

#### 2.5.5. Scanning Electron Microscopy (SEM)

The microstructures of AP‐P and AP‐Se were examined by a scanning electron microscope (JEOL JSM‐6390LV, Tokyo, Japan). Gold was sprayed and sputter‐coated into the powder samples. The specimens were examined under high vacuum conditions with the 500× and 3000× magnification.

#### 2.5.6. Simultaneous Thermal Analysis (STA)

The STA of AP‐P and AP‐Se was evaluated by a synchrotron thermal analyzer (PerkinElmer, United States). AP‐P and AP‐Se powders were mixed in an alumina crucible, with the empty crucible serving as a reference adjustment baseline. The temperature range was 30°C~900°C, with temperature increase rate of 10°C/min and a nitrogen flow rate of 25 mL/min.

### 2.6. In Vitro Activities

#### 2.6.1. Antioxidant Activities

The DPPH^+^ radical scavenging activities of AP‐P and AP‐Se were determined by the method of Zhu et al. with slight modifications [18]. First, AP‐P, AP‐Se, and VC solution of 0.075, 0.15, 0.3, 0.6, and 1.2 mg/mL was made. Then, sample solution and 0.1 mM DPPH^+^ ethanol solution was mixed in a ratio of 1:1, which was vigorously agitated and reflected at 25°C in the dark for 30 min before measuring its absorbance at 517 nm. VC served as a positive control. The DPPH^+^ scavenging efficiency was computed as follows:

Scavenging activity %=A0−Ai+Aj/A0×100%

where A_0_ is the absorbance value (Abs) of the control (ultrapure water instead of the sample), A_i_ is the Abs of the sample solution and DPPH^+^‐ethanol solution. A_j_ is the Abs of sample solution only (ethanol instead of DPPH^+^‐ethanol solution).

The ABTS^+^ radical scavenging activities of AP‐P and AP‐Se were measured with reference to Zhu et al. with some modifications [18]. Briefly, AP‐P, AP‐Se, and VC were dissolved at concentrations of 0.0375, 0.075, 0.15, 0.3, and 0.6 mg/mL, respectively. Seven millimolars of ABTS^+^ and 2.6 mM K_2_S_2_O_8_ were mixed in the ratio of 1:1 and stored away from light for 12 h; then, Abs of mixture was adjusted to 0.7 ± 0.02 by PBS (pH 7.4) to obtain ABTS^+^ worked solution prior to the experiment. Two milliliters of sample solution was combined with 2 mL of ABTS^+^ worked solution, mixed thoroughly and kept in the dark at 25°C for 30 min, followed by measuring its Abs at 734 nm. VC was served as a positive control. The ABTS^+^ clearance efficiency was computed as follows:

Scavenging activity %=A0−Ai+Aj/A0×100%

where A_0_ is the Abs of the control (ethanol instead of sample), A_i_ is the Abs of the sample solution, and A_j_ is the Abs of sample solution only (ethanol instead of ABTS^+^‐ethanol solution).

The hydroxyl radical scavenging activities of AP‐P and AP‐Se were as detected based on the methods of Ye et al., with slight modifications [5]. Briefly, AP‐P, AP‐Se, and VC were dissolved at concentrations of 0.075, 0.15, 0.3, 0.6, and 1.2 mg/mL, respectively. Six millimolars of FeSO_4_, 6 mM salicylic acid, and 6 mM H_2_O_2_ solutions were prepared in advance. In the experiment, 1 mL sample solution was mixed with 1 mL FeSO_4_, 1 mL of salicylic acid, and 1 mL H_2_O_2_. The mixture was vigorously agitated and maintained in the dark at 37°C for 30 min; then, its Abs at 520 nm was measured. VC was served as a positive control. The hydroxyl scavenging activity was computed as follows:

Scavenging activity %=A0−Ai+Aj/A0×100%

where A_0_ is the Abs of the control (ultrapure water instead of the sample), A_i_ is the Abs of the sample solution, and A_j_ is the Abs of the sample solution (ultrapure water instead of the H_2_O_2_).

The reducing power was determined by the approach of Ye et al., with some modifications [5]. Briefly, AP‐P, AP‐Se, and VC were dissolved at concentrations of 0.0375, 0.075, 0.15, 0.3, and 0.6 mg/mL, respectively. PBS buffer (0.2 M; pH 6.6), 1% potassium ferricyanide, 10% trichloroacetic acid, and 0.1% FeCl_3_ were prepared. PBS buffer (2.0 mL) and 2.0 mL potassium ferricyanide solution were added to the 2.0 mL sample solution, which was subsequently incubated in a water bath at 50°C for 20 min. Then, 2.5 mL of trichloroacetic acid solution was added and the sample was centrifuged at 3000 g for 10 min. Following by adding 0.5 mL FeCl_3_ solution and 1.0 mL ultrapure water to 1.0 mL sample supernatant. The absorbance of the reaction mixture was detected at 700 nm. VC was served as a positive control. The higher the absorbance value of the reaction mixture, the larger its reduction potential.

#### 2.6.2. Intestinal Permeability

Intestinal permeability of AP‐Se and Se in the gastrointestinal tract was assessed by simulating in vitro digestion [19]. The in vitro digestion procedure was performed as described in a previous study [20].

After a gastric phase of digestion, some of the digested solution was transferred to a dialysis bag (molecular weight cutoff: 7000 Da) to simulate the intestinal environment. After intestinal digestion, the Se content in the solution outside dialysis bag was determined and the intestinal permeability was calculated as follows:

Permeability=C1/C2×100%

where C_1_ is the Se concentration of the solution outside the dialysis bag; C_2_ is the Se concentration of the digestive solution.

#### 2.6.3. Cytotoxicity Evaluation

Cell Culture. The cell culture medium was DMEM supplemented with 20% fetal bovine serum and 1% double antibiotic. Cells were incubated at 37°C with a 5% carbon dioxide concentration, and were subcultured with 0.25% trypsin EDTA at a ratio of 1: 2.

Cytotoxicity Assay. The CCK‐8 test was used to evaluate the cytotoxic of AP‐Se. Cell lines in logarithmic growth phase were inoculated in 96‐well plates at a density of 2 × 10^4^ cells per well. The cells were washed twice with PBS and reconstituted with culture media containing AP‐Se chelate (the contained Se concentration was 55, 41.25, 27.5, 5.5, and 1.1 *μ*g/mL), and the same Se content of sodium selenite was added as the control groups. After 24 h of incubation, the cells were washed twice with PBS and replaced with a medium containing 10% CCK‐8 solution. After 1 h of incubation, the absorbance was measured at 450 nm.

### 2.7. Statistical Analysis

BBD was analyzed and plotted by Design‐Expert software (Version 13.0.1) and Origin software (Version 2021). Analysis of variance was studied by ANOVA and means were compared with Duncan’s new multiple range test with SPSS software (Version 27.0.1). All data were presented as the mean ± standard deviations (*n* = 3). The significance of the differences was defined as the 5% level (*p* < 0.05).

## 3. Results and Discussion

### 3.1. Optimization of Preparation Conditions for AP‐Se

#### 3.1.1. Results of Single Factor Experiments

In the optimization of chelation conditions for AP‐Se, the effects of five single parameters (peptide concentration, peptide‐Se mass ratio, temperature, pH, and time) on Se chelating ability were explored. As demonstrated in Figure 1a, the Se chelating capacity increased with peptide concentration, peaking at 6%. Se chelating ability did not elevate with continuously increased peptide concentration, for avoiding result waste of resources, the peptide concentration was set at 6% in following trials.

Figure 1Major factors affecting Se chelating capacity (mg/g), (a) Peptide concentration (%), (b) peptide‐selenium mass ratio (g/g), (c) chelation temperature, (d) chelation pH, (e) chelation time, Response surface plots and contour plots (f) showing the effect of peptide‐selenium mass ratio (X_1_), chelation temperature (X_2_), chelation pH (X_3_) on Se chelating capacity, different letters indicate significant differences (*p* < 0.05).

**Figure:**
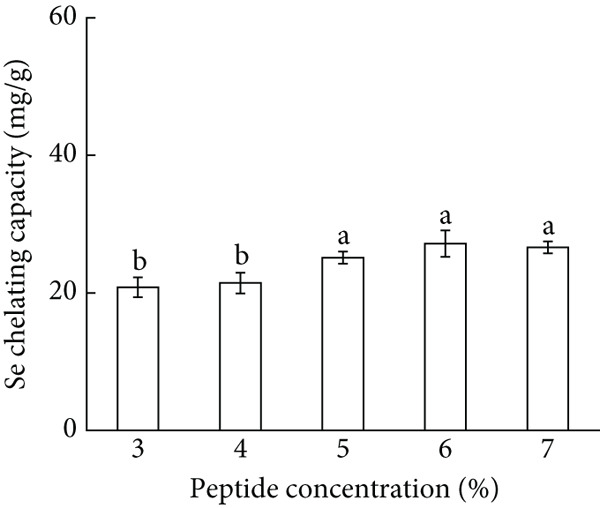
(a)

**Figure:**
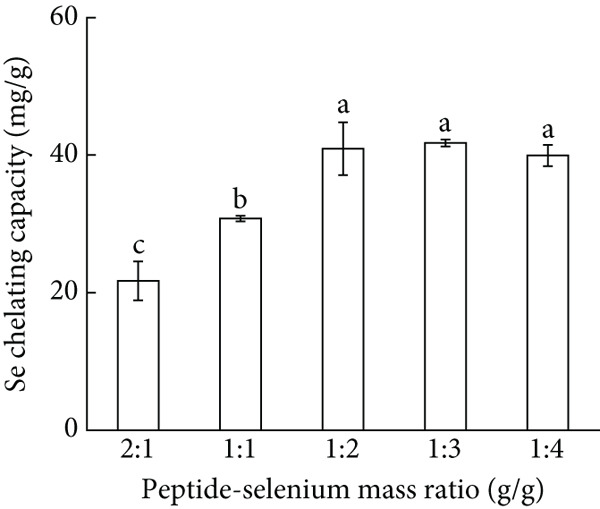
(b)

**Figure:**
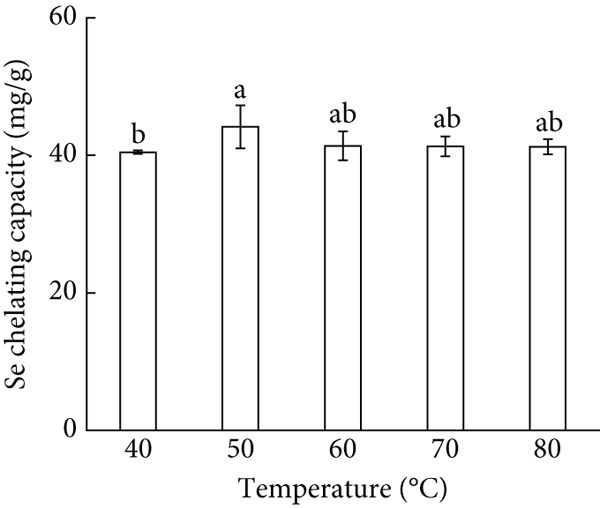
(c)

**Figure:**
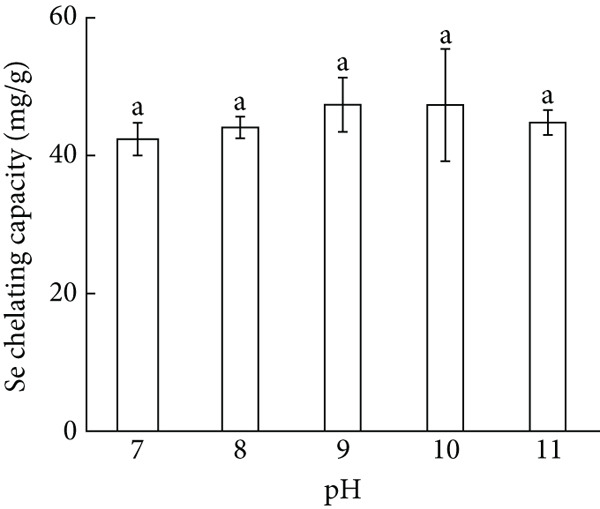
(d)

**Figure:**
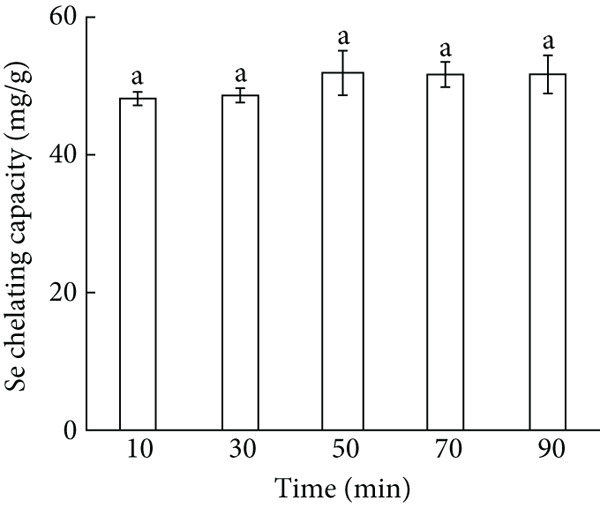
(e)

**Figure:**
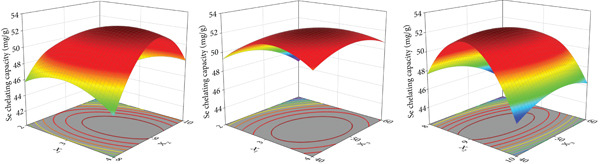
(f)

In the process of chemical reaction, the relative ratios of reactants may constitute an influence on the binding of some spatial bonding sites. As shown in Figure 1b, the maximum Se chelating capacity reached 44.02 mg/g when the peptide‐Se mass ratio was 1:3, whereas Se chelating capacity declined with the increased peptide‐Se mass ratio. Therefore, a mass ratio of 1:3 was chosen for subsequent trials.

Figure 1c exhibited the influence of chelation temperature on Se chelating capacity. The maximum Se chelating capacity (47.43 mg/g) was achieved when the temperature reached 50°C. Appropriate temperature can stimulate the thermal movement of molecules, inducing the peptide chains to unfold and exposing more sites that can be chelated with Se, which makes the chelation of AP‐P and Se easier while also promotes the chelation reaction [21].

pH value is one of the crucial factors to affect the Se chelating capability. At lower pH, the H^+^ in the solution binds to the carboxylate in AP‐P and some amino acids in the peptide chain are protonated, which is not conducive to the chelation of AP‐P with Se and thus reduced the chelating capacity. At pH 9, the chelating capacity increased to 47.38 mg/g, demonstrating that the weakly alkaline circumstance was conducive to the chelation reaction. Continuously raised pH led to a gradual decline in the chelating capacity, which might be due to the formation of side reactions such as peptide decomposition in the system, that is detrimental to the chelation reaction (Figure 1d).

The effect of chelation time on the Se chelating capacity was shown in Figure 1e. With the extension of reaction time, the Se chelating capacity increased continuously and reached the maximum value (51.92 mg/g) at 50 min. Long chelating time leads to a decrease in the stability of the system and a decrease in the amount of Se chelating capacity [22]. Thus, the chelating time was determined to be 50 min in the BBD experiments.

#### 3.1.2. Results of BBD

The three factors with the highest influence on Se chelating capacity, peptide‐Se mass ratio, pH value, and temperature, were chosen for optimizing BBD chelation conditions. A peptide concentration of 6% and a chelation duration of 70 min were determined, and 17 sets of experiments were set and carried out. Following multiple regression fitting, the quadratic regression equation for peptide‐selenium mass ratio, pH, and temperature was found as: $\text{Y}\;\left(\text{Se}\;\text{chelating}\;\text{capability}\right)=53.381.280.130.551.740.771.051.965.311.18+{\text{X}}_{1}-{\text{X}}_{2}-{\text{X}}_{3}+{\text{X}}_{1}{\text{X}}_{2}+{\text{X}}_{1}{\text{X}}_{3}+{\text{X}}_{2}{\text{X}}_{3}-{{\text{X}}_{1}}^{2}-{{\text{X}}_{2}}^{2}-{{\text{X}}_{3}}^{2}$, X_1_: peptide‐Se mass ratio, X_2_: temperature, X_3_: pH.

Table 2 displayed the results of the ANOVA of the RSM regression model. The values of *R* ^2^ and Adj *R* ^2^ were 0.9873 and 0.9710 for the obtained regression model (*p* < 0.0001), indicating that the overall quadratic response surface model was highly significant and important [23]. The lack of fit term *p* = 0.1015 > 0.05 indicated that the misfit term was not significant, indicating that the equation can well describe the relationship between different AP‐Se chelating conditions. X_1_ and X_3_ had considerable influence on response values, while the X_1_ had the greatest effect followed by X_2_ and X_3_. The largest quadratic term interaction was discovered between X_1_X_2_, i.e., the peptide‐Se mass ratio and pH. Following that was X_2_X_3_, which represented the interaction between pH and temperature, and finally X_1_X_3_, which represented the relatively minor interaction between peptide‐selenium mass ratio and temperature.

**Table 2:** Analysis of variance (ANOVA) for experimental results.

**Source**	**Sun of squares**	**DF**	**Mean square**	**F** ** value**	**p** ** value**
Model	185.01	9	20.56	60.59	< 0.0001
X_1_	13.10	1	13.10	38.60	0.0004
X_2_	0.14	1	0.14	0.41	0.5412
X_3_	2.44	1	2.44	7.18	0.0316
X_1_X_2_	12.09	1	12.09	35.62	0.0006
X_1_X_3_	2.36	1	2.36	6.59	0.0336
X_2_X_3_	4.39	1	4.39	12.95	0.0088
X_1_^2^	16.17	1	16.17	47.65	0.0002
X_2_^2^	118.66	1	118.66	349.73	< 0.0001
X_3_^2^	5.91	1	5.91	17.41	0.0042
Residual	2.37	7	0.34		
Lack of Fit	1.08	3	0.60	4.15	0.1015
Pure error	0.58	4	0.14		
Cor total	187.39	16			
		*R* ^2^ = 0.9873	Adj *R* ^2^ = 0.9710		

The response surface plots in Figure 1f depicted the interplay of the two parameters on Se chelating capacity. The inclination of the top spatial plane in the image represented the degree to which the relevant factor influences the response value, whereas the eccentricity of the ellipse in the lower contour plot represented the amount of the interaction between the two elements. The multifactor fitting investigation indicated the most effective chelation process for AP‐Se at 1:3.32 peptide‐Se mass ratio, 9.03 pH, and 48.8°C. The maximal theoretical value of Se chelating capacity under these conditions was 52.61 mg/g. Finally, the peptide‐Se mass ratio of 1:3.3, pH 9.0, and temperature 49°C was selected as the optimum condition for AP‐Se chelation actually. Under this condition, the measured Se chelating capacity was 52.78 ± 0.74 mg/g. The validation result was similar to the prediction result. Then, the AP‐Se was made by the optimized conditions and freeze‐dried for all the following experiments.

### 3.2. AP‐Se Structural Characterization

#### 3.2.1. UV Spectroscopy

Figure 2a displayed the UV absorption spectra of AP‐P and AP‐Se in the 240~320 nm range. The UV absorption spectra of AP‐Se and AP‐P differed in absorbance strength and position, with AP‐Se exhibiting a stronger absorption peak near the wavelength of 285 nm, which may be due to AP‐Se’s stronger light‐absorbing structure and the chelated selenium with AP‐P valence electron transition. Simultaneously, ligand binding probably induces electron transitions within the ligand, and the change in valence electron transition causes a change in UV absorption [4]. Thus, chelation with Se changed the structure of AP‐P.

Figure 2Structural characteristics of AP‐P and AP‐Se: (a) UV spectrum, (b) FTIR spectrum, (c) CD spectroscopy, (d) secondary structure content, different letters indicate significant differences within groups. (*p* < 0.05), different “*” quantities indicate significant differences between groups (*p* < 0.05). (e) X‐ray diffraction, (f) scanning electron micrograph (a‐AP‐P 500×; b‐AP‐P 3000×; c‐AP‐Se 500×; d‐AP‐Se 3000×), (g) simultaneous thermal analysis (AP‐P), (h) simultaneous thermal analysis (AP‐Se).

**Figure:**
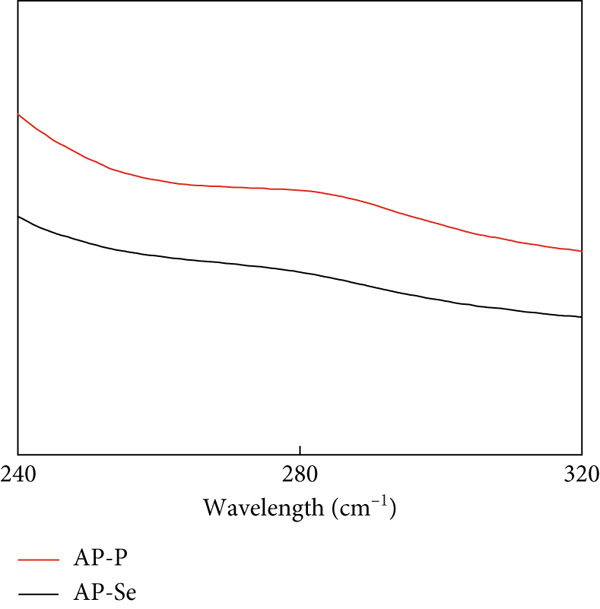
(a)

**Figure:**
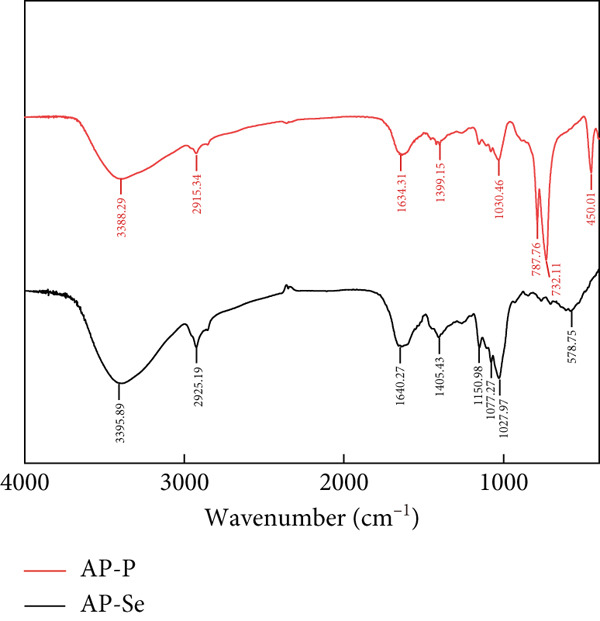
(b)

**Figure:**
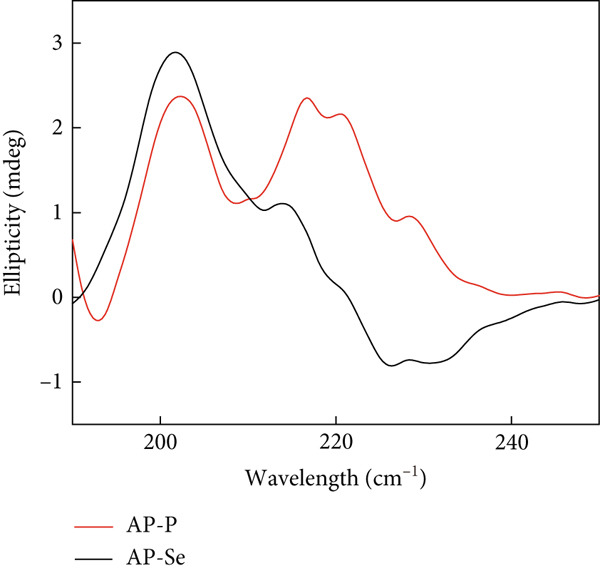
(c)

**Figure:**
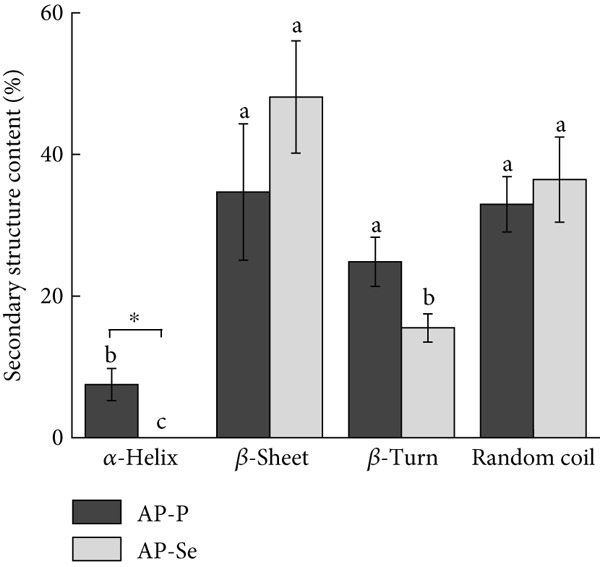
(d)

**Figure:**
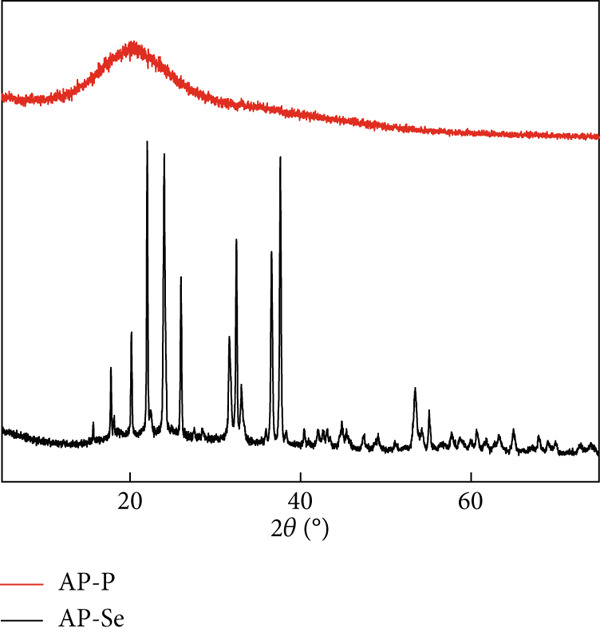
(e)

**Figure:**
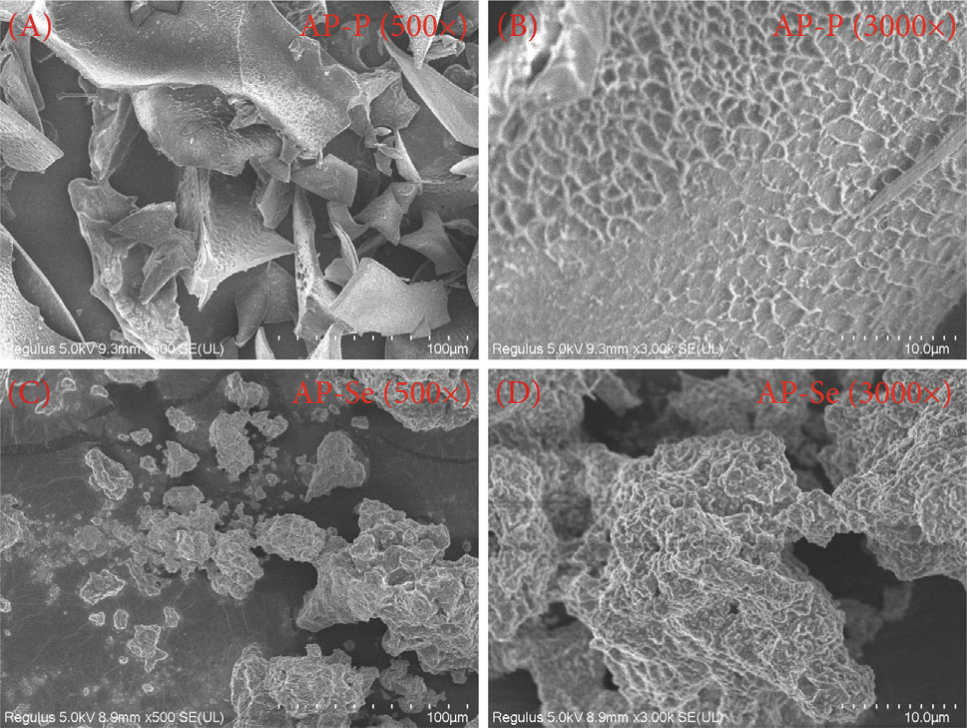
(f)

**Figure:**
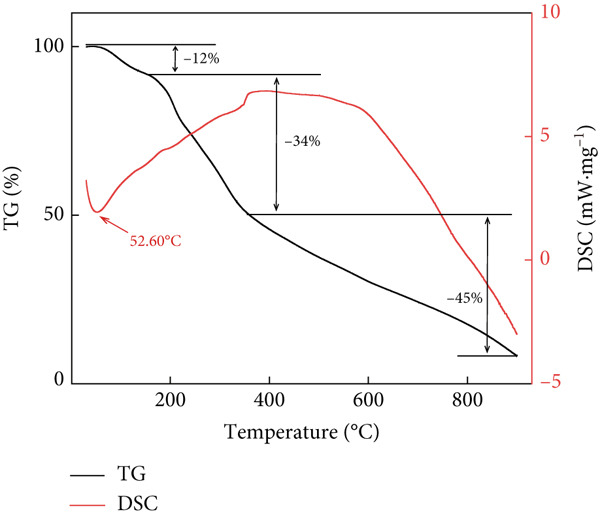
(g)

**Figure:**
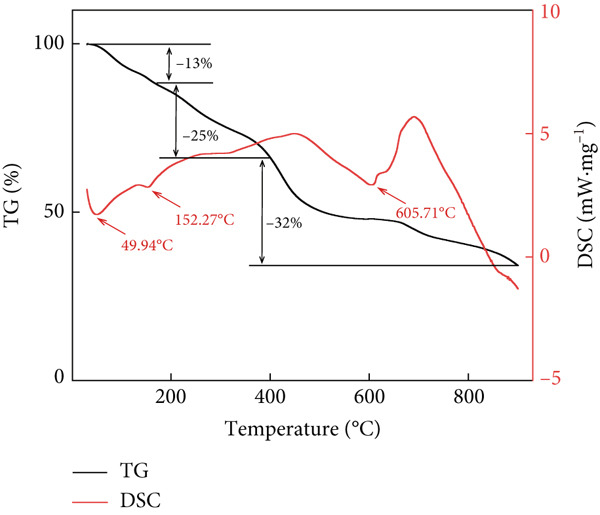
(h)

#### 3.2.2. FTIR Spectroscopy

In Figure 2b, the FTIR spectra of AP‐P and AP‐Se in the range of 4000~400 cm^−1^ was displayed. The FTIR spectra of AP‐P after chelating with Se were red‐shifted overall and the strength of the peaks before 1300 cm^−1^ increased. In particular, a large absorption peak with the overlap of ‐OH and ‐NH stretching vibrational frequencies occurred at 3388 cm^−1^, but when the wave number grew to 3395 cm^−1^ in the spectrogram of AP‐Se, the peak became narrower and more intense. This suggested that Se ions may react with the ‐OH or ‐NH groups [24]. Chelation with Se made the absorption peak of ‐NH on ‐NH‐C‐O at 1030 cm^−1^ red shifted, causing the peak intensity to decrease and narrow. It showed that a portion of the ‐NH‐C=O may undergo chelation process with selenium ions. In conclusion, it was assumed that the N‐terminal amino group and C‐terminal carboxyl group of AP‐P had chelation reactions with selenium ion coordination, which was consistent with the findings of Ye et al. [5]. Thus, it was possible to conclude that AP‐P chelated with selenium ions, resulting in the formation of a new complex.

#### 3.2.3. CD Spectroscopy

CD spectroscopy is a method for the research of the protein or peptide secondary structure (including ligand binding of peptides) in the “far‐UV” spectral region (190~260 nm) [25, 26]. Figure 2c,d revealed that AP‐P’s secondary structure was primarily consisted of *β*‐sheet (34.7%), *β*‐turn (27.3%), and random coil (32.95%), with less *α*‐helix (7.5%). When AP‐P was coupled with selenium, the *α*‐helix structure disappeared, *β*‐folding increased to 48.1%, and *β*‐turn dropped to 15.5%. However, no significant alterations were seen in the random coil. The findings showed that Se chelating caused peptide folding and the formation of a more compact secondary structure. These were comparable with the findings of Qin et al. [27].

#### 3.2.4. XRD Analysis

The XRD can reflect the difference in the molecular microstructure after chelation of organic ligands and ions [28]. Figure 2e exhibited the XRD patterns of AP‐P and AP‐Se chelates. The diffraction absorption peaks of AP‐P emerged near 2*θ* of 20°. The strength of the absorption peaks was smaller and the background was large, indicating that AP‐P was amorphous structures. Chelation with selenium ions resulted in significant changes in the number, angle, and relative intensity of the AP‐Se peaks, revealing that the AP‐Se and AP‐P had different compositions and structures. After the formation of the chelate, AP‐Se displayed many strong and weak peaks. Several of the diffraction peaks were high and sharp, and the grain size became smaller, indicating that AP‐Se formed a better‐shaped crystal structure. This was consistent with the analytical conclusions derived from FTIR, which suggested that the crystal structure of AP‐P changed when the carboxyl, amino and selenium ions combined to form a ligand bond. This was similar to the findings of Xiong et al. [7].

#### 3.2.5. SEM

The SEM imaging of AP‐P was shown in Figure 2f(a,b). AP‐P had a flat lamellar structure with irregular arcs evident at 3000× magnification. As demonstrated in Figure 2f(c,d), the microstructure of AP‐Se was more compact than AP‐P, the binding reaction probably produced agglomerates with more folds and crystalline structure, which was similar to sea cucumber ovum hydrolysate–calcium complex [21]. Changes in microstructure of the particles formed after chelation indicated that AP‐P bound to Se ions to form dense particles. It could be the interactions between the metal ions and peptides to promote peptides polymerization, and hydrogen bonding in water molecules also led to the aggregation of carboxyl and amino groups in the peptide chain [4]. The above findings suggested that the altered structure of AP‐P provided binding sites for Se ions, resulting in the formation of AP‐Se.

#### 3.2.6. Simultaneous Thermal Analysis (STA)

Physicochemical change processes such as dehydration, evaporation, decomposition or polymerization, crystallization, and adsorption, can occur during the heating process, which is accompanied by heat absorption or exotherm. Therefore, the mass‐temperature dependence of the substance was investigated by STA and its thermal stability was further studied. The TG‐DSC curve of AP‐P was shown in Figure 2g, AP‐P exhibited three stages of weight loss during the heating process. The first stage was occurred during 30~180°C, the mass decreased by 12% and an endothermic peak appeared at 52.6°C, which may be caused by the C‐N bond in AP‐P [29]. The second stage weightlessness of AP‐P reached 34% in range of 180°C–400°C, which mainly caused by the degradation of unstable polymer, indicating that its chemical structure was decomposed in this temperature range. The third mass loss stage was a continuous slight devolatilization, and AP‐P produced oxygenated compounds and carbon‐containing residues with a mass residue of 8.22% when temperature reached 900°C. Whereas, multiple endothermic peaks were observed in the DSC curves of AP‐Se at 49.94°C, 152.27°C and 605.71°C (Figure 2h), which demonstrated that there was an inhomogeneous calorimetric response of AP‐Se. Concurrently, the TG curve of AP‐Se showed a flat downward trend with a mass loss of 70%. This suggested that the chelation of Se to AP‐P caused a significant increase in the thermal decomposition temperature and a simultaneous decrease in mass loss, which indicated that AP‐Se had a favorable thermal stability.

### 3.3. In Vitro Biological Activities

#### 3.3.1. Antioxidant Activities

For investigating the antioxidant capacities of AP‐P and AP‐Se, free radical scavenging and reducing capacities were determined, respectively. As shown in Figure 3a, the DPPH^+^ clearance rates of AP‐P and AP‐Se was gradually enhanced with increased concentration. Compared with AP‐Se, AP‐P had a stronger clearance of DPPH^+^ and the disparity increased with higher concentration, at a sample concentration of 0.25 mg/mL, the DPPH^+^ clearance rates of AP‐P was observed to be 2.2 times that of AP‐Se (*p* < 0.05). In contrast, AP‐P and AP‐Se both had superior ABTS^+^ clearance rates, which were comparable with VC after 0.15 mg/mL (Figure 3b). Overall, both AP‐P and AP‐Se exhibited higher DPPH^+^ and ABTS^+^ clearance rates than bovine bone collagen peptides and their metal ion chelates (Ca^2+^, Fe^2+^, Zn^2+^, Mg^2+^, and Cu^2+^) [30]. The antioxidant effect of Se is mediated by modulating oxygen radicals and antioxidant enzyme activities [31]. This may be one of the reasons why the ˙OH clearance rate of AP‐Se was higher than AP‐P (Figure 3C), and this difference was gradually apparent with elevated concentration. At a sample concentration of 1.20 mg/mL, the OH clearance rate of AP‐Se was found to be 1.9 times that of AP‐P (*p* < 0.05). Moreover, the addition of Se ions altered the structure of the peptide chain, exposing more amino acids with antioxidant activity [32]. Similarly, He et al. [33] demonstrated that the antioxidant activity of oat peptides was increased by binding ferrous ions through *in vivo* experiments. Although the reducing capacity of AP‐P and AP‐Se for Fe^3+^ was significantly weak than VC, but it still displayed an approximate dose‐dependent correlation (Figure 3d). Moreover, at a series of concentrations, the Fe^3+^reducing capacity of AP‐P is 1.17 to 1.85 times that of AP‐Se (*p* < 0.05).

Figure 3Antioxidant activity of AP‐P and AP‐Se (compared with vitamin C): (a) DPPH^+^ clearance rate, (b) ABTS^+^ clearance rate, (c) OH clearance rate, (d) reducing capacity, different lowercase letters indicate significant differences between groups (*p* < 0.05), different uppercase letters indicate significant differences within groups (*p* < 0.05).

**Figure:**
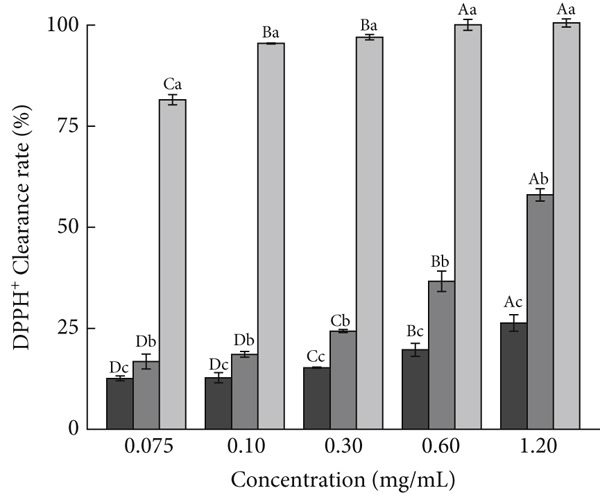
(a)

**Figure:**
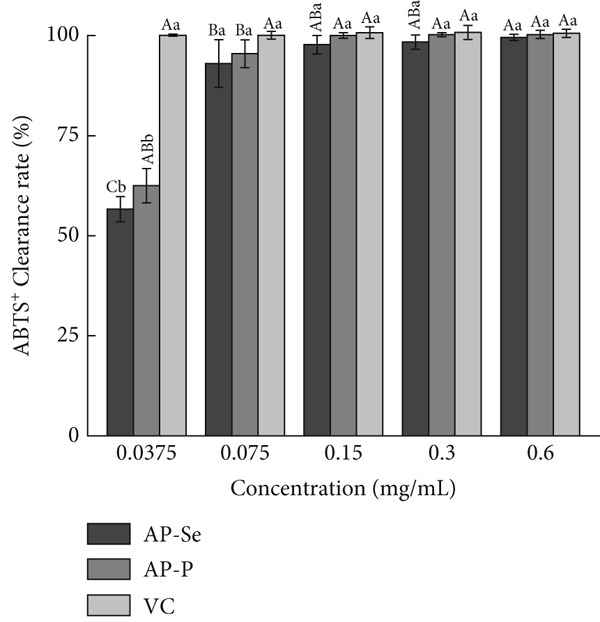
(b)

**Figure:**
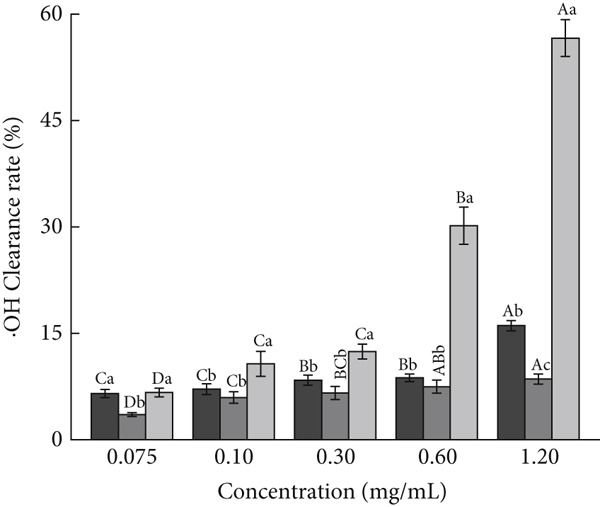
(c)

**Figure:**
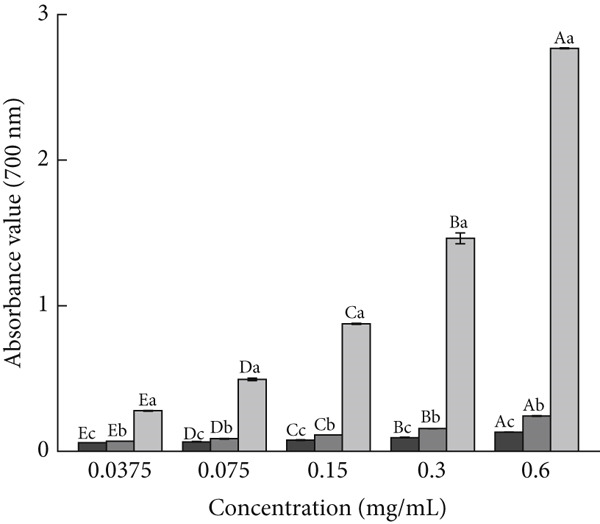
(d)

#### 3.3.2. Intestinal Permeability

The digestive environment, particularly various gastrointestinal proteases and peptidases, could affect the bioavailability of Se supplements [34]. Therefore, the digestive properties of Se in the gastrointestinal tract were explored, and the intestinal permeability of AP‐Se and sodium selenite in the gastrointestinal tract was compared by simulating *in vitro* digestion.

The intestinal permeability of Se in AP‐Se and sodium selenite during simulated intestinal digestion was shown in Figure 4a. The Se permeability of sodium selenite raised from 11.69% to 18.04% as time increased and reached the basic equilibrium at 180 min. The Se permeability of AP‐Se increased dramatically from 4.5% to 54.94%. Following simulated intestinal digestion, the Se permeability of AP‐Se was found to be three times that of sodium selenite (*p* < 0.05). It suggested that AP‐Se may be resistant to the environmental conditions of intestine, and can be further decomposed into small molecules by gastric/pancreatic proteases and thus been absorbed after entering intestine. This assumption was testified in Hou et al.’s [35] research that the Wheat protein peptides selenium chelates (WPH‐Se) precipitates in the stomach can be redissolved in the intestines, where the intestinal environment promotes its release and absorption. This suggests that peptides chelation may be beneficial for enhancing selenium absorption in the human body. In conclusion, AP‐Se has been demonstrated to efficiently maintain and release Se that is highly bioavailable following digestion, thus further emphasizing its potential as a beneficial dietary supplement that can improve the body’s absorption of Se.

Figure 4(a) Intestinal permeability of AP‐Se (compared with Na_2_SeO_3_), (b), (c) and (d) cytotoxicity of AP‐Se (compared with Na_2_SeO_3_), different “∗” quantities indicate significant differences (*p* < 0.05).

**Figure:**
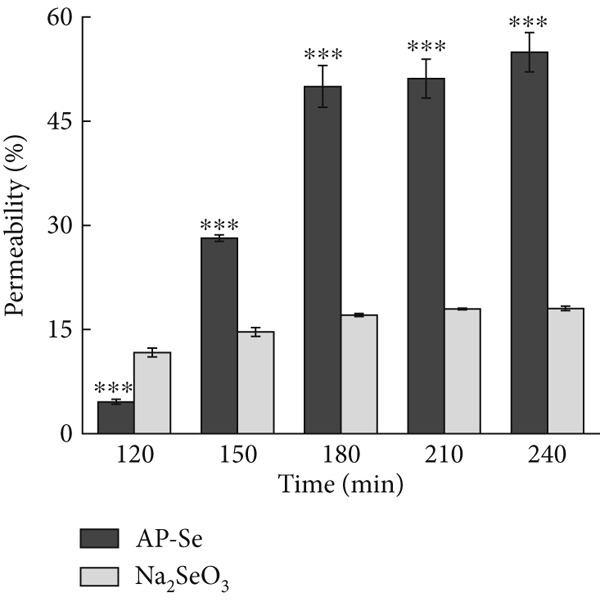
(a)

**Figure:**
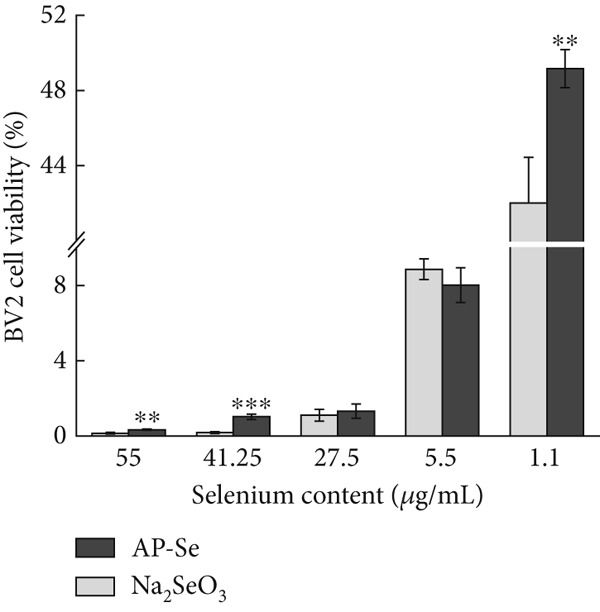
(b)

**Figure:**
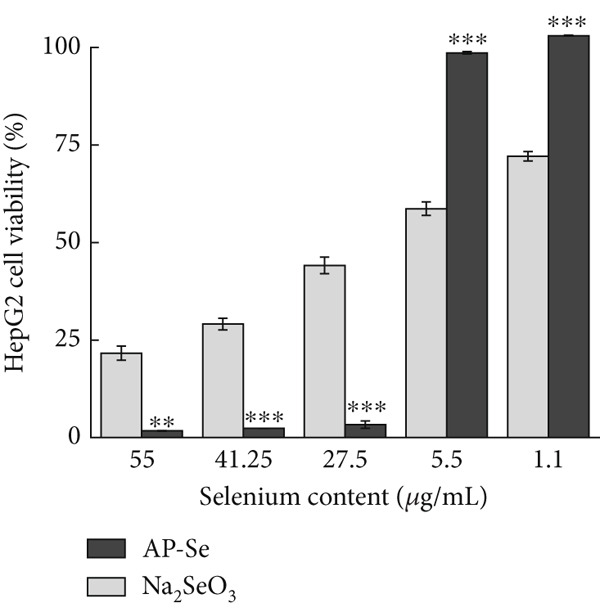
(c)

**Figure:**
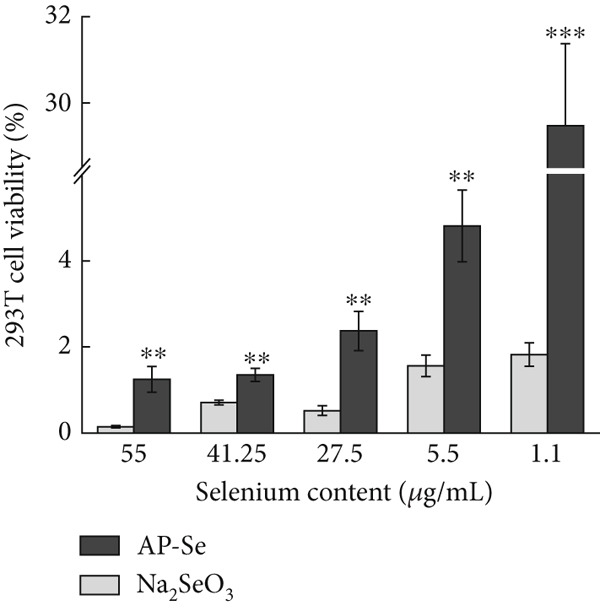
(d)

#### 3.3.3. Cytotoxicity Evaluation

The cytotoxicity evaluation was carried out by CCK‐8 assay on mouse microglial BV2 cells, human embryonic kidney 293T cells and human hepatocellular carcinoma HepG2 cells. During the test, the water‐soluble tetrazolium salt (WST‐8) in CCK‐8 was reduced to the yellow‐colored water‐soluble formazan product by dehydrogenase enzyme in cellular mitochondria in the presence of electron carrier dimethyl sulfate (1‐Methoxy PMS), and the amount of generated filth was proportional to the number of living cells. The absorbance value measured at 450 nm can indirectly reflect the number of viable cells [36]. As shown in Figure 4b,c,d, the proliferation of cells was inhibited by both AP‐Se and sodium selenite. Cell survival generally increased with decreased sample concentration, but sodium selenite exhibited greater cytotoxicity than AP‐Se at all tested concentrations. The toxicities of AP‐Se and sodium selenite to HepG2 cells are significantly different. With sodium selenite being significantly less toxic than AP‐Se at high concentrations, but sodium selenite showed higher cytotoxicity than AP‐Se at low concentrations. For the 293T cells, the cytotoxicity of AP‐Se was significantly lower than sodium selenite. In summary, AP‐Se was overall less toxic to cells than sodium selenite, and the three cells demonstrated higher cell viability in the presence of low drug concentrations. For improving the AP‐Se application, further in‐depth studies are needed on the cellular uptake mechanism and toxicity mechanism of AP‐Se. Similarly, *Grifola frondosa* protein hydrolysate peptide selenium chelate has higher biological activity and lower toxicity compared with sodium selenite [7]. This suggested that AP‐Se had the potential to be developed as a novel Se supplement with low toxicity.

## 4. Conclusion

The optimal chelation process of AP‐P with sodium selenite was determined by one‐way test and RSM, and the structure and bioactivity of AP‐Se were investigated. Results implied that the optimized chelation conditions achieved Se chelating capacity to 52.78 ± 0.74 mg/g. AP‐P chelated with selenium ions, resulting in the formation of a new chelate. The chelation reaction folded and curled the peptide chains in AP‐P, resulting in a stable crystal structure of AP‐Se. Furthermore, AP‐Se had superior antioxidant capacity. Among these, AP‐P exhibited DPPH^+^ clearance rates 2.2 times (1.20 mg/mL) (*p* < 0.05) higher than AP‐Se and Fe^3+^reducing capacity 1.85 times greater (0.60 mg/mL) (*p* < 0.05). Both compounds demonstrated comparable and ABTS^+^ clearance rates (0.15~0.60 mg/mL), while the ˙OH clearance rate of AP‐Se was 1.9 times (1.20 mg/mL) (*p* < 0.05) higher than that of AP‐P. Compared with sodium selenite, AP‐Se was found to have superior intestinal permeability, with the AP‐Se selenium permeation rate three times greater than that of sodium selenite (*p* < 0.05). Meanwhile, AP‐Se has lesser cytotoxicity than sodium selenite. The research findings have offered a scientific foundation for developing novel Se supplements. However, the mechanism of chelate metabolism in mammals is yet unknown and still needs to be further researched.

## Ethics Statement

This study does not involve any human or animal testing.

## Conflicts of Interest

The authors declare no conflicts of interest.

## Author Contributions

**Wen-Lu Wei:** conceptualization, conducted the research and data analysis, writing the original draft. **Wen-Jun Wang:** supervised the research, review, and editing. **Chuan-Long Yu:** conducted the research and data analysis. **Hui Chen** and **Su-Yun Lin:** date analysis and editing. **Ling-Li Chen:** conceived and designed the research, supervision, writing, review and editing, and funding acquisition.

## Funding

This study was supported by National Natural Science Foundation of China, 32460223; Jiangxi Provincial Natural Science Foundation, 20242BAB20324.

## Data Availability

Data will be made available on request.

## References

[1] Steinbrenner H. , Interference of Selenium and Selenoproteins With the Insulin-Regulated Carbohydrate and Lipid Metabolism, Free Radical Biology and Medicine. (2013) 65, 1538–1547, 10.1016/j.freeradbiomed.2013.07.016, 2-s2.0-84890438398, 23872396. 23872396

[2] Zhang J. , Zhou H. , Li H. , Ying Z. , and Liu X. , Research Progress on Separation of Selenoproteins/Se-Enriched Peptides and Their Physiological Activities, Food & Function. (2021) 12, no. 4, 1390–1401, 10.1039/D0FO02236E, 33464257. 33464257

[3] Xie M. , Sun X. , Li P. , Shen X. , and Fang Y. , Selenium in Cereals: Insight Into Species of the Element From Total Amount, Comprehensive Reviews in Food Science and Food Safety. (2021) 20, no. 3, 2914–2940, 10.1111/1541-4337.12748, 33836112. 33836112

[4] Tang L. , Gao X. , Sun Y. , Tian T. , and Li S. , Preparation Process Optimisation, Structural Characterisation and Stability Analysis of Sika Deer Blood–Selenium Chelate, Animal Production Science. (2023) 63, no. 13, 1349–1360, 10.1071/AN22415.

[5] Ye Q. , Wu X. , Zhang X. , and Wang S. , Organic Selenium Derived From Chelation of Soybean Peptide-Selenium and its Functional properties In Vitro and In Vivo, Food & Function. (2019) 10, no. 8, 4761–4770, 10.1039/C9FO00729F, 2-s2.0-85070812942. 31309961

[6] Zhao X. , Zhao Q. , Chen H. , and Xiong H. , Distribution and Effects of Natural Selenium in Soybean Proteins and its Protective Role in Soybean *β*-Conglycinin (7S Globulins) Under AAPH-Induced Oxidative Stress, Food Chemistry. (2019) 272, 201–209, 10.1016/j.foodchem.2018.08.039, 2-s2.0-85051498068, 30309533. 30309533

[7] Xiong Y. , Chen Z.-H. , Zhang F.-L. , Yu Z.-Y. , Liu B. , Zhang C. , and Zhao L.-N. , A Specific Selenium-Chelating Peptide Isolated From the Protein Hydrolysate of *Grifola frondosa* , RSC Advances. (2021) 11, no. 17, 10272–10284, 10.1039/D0RA10886C, 35423524. 35423524 PMC8695590

[8] Kotynia A. , Wiatrak B. , Kamysz W. , Neubauer D. , Jawień P. , and Marciniak A. , Cationic Peptides and Their Cu(II) and Ni(II) Complexes: Coordination and Biological Characteristics, International Journal of Molecular Sciences. (2021) 22, no. 21, 12028, 10.3390/ijms222112028, 34769458. 34769458 PMC8584440

[9] Luo J. , Zhou Z. , Yao X. , and Fu Y. , Mineral-Chelating Peptides Derived From Fish Collagen: Preparation, Bioactivity and Bioavailability, LWT. (2020) 134, 110209, 10.1016/j.lwt.2020.110209.

[10] Cai F.-F. , Wu R. , Song Y.-N. , Xiong A.-Z. , Chen X.-L. , Yang M.-D. , Yang L. , Hu Y. , Sun M.-Y. , and Su S.-B. , Yinchenhao Decoction Alleviates Liver Fibrosis by Regulating Bile Acid Metabolism and TGF-*β*/Smad/ERK Signalling Pathway, Scientific Reports. (2018) 8, no. 1, 15367, 10.1038/s41598-018-33669-4, 2-s2.0-85055076256, 30337590. 30337590 PMC6194075

[11] Hung H.-Y. and Kuo S.-C. , Recent Studies and Progression of Yin Chen Hao (茵陳蒿 Yīn Chén Hāo), a Long-Term Used Traditional Chinese Medicine, Journal of Traditional and Complementary Medicine. (2013) 3, no. 1, 2–6, 10.4103/2225-4110.106533, 2-s2.0-84894540157, 24716150. 24716150 PMC3924980

[12] Gao Q. , Zhao X. , Yin L. , Zhang Y. , Wang B. , Wu X. , Zhang X. , Fu X. , and Sun W. , The Essential Oil of *Artemisia capillaris* Protects Against CCl4-Induced Liver Injury In Vivo, Revista Brasileira de Farmacognosia. (2016) 26, no. 3, 369–374, 10.1016/j.bjp.2016.01.001, 2-s2.0-84975089826.

[13] Okuno I. , Uchida K. , Kadowaki M. , and Akahori A. , Choleretic Effect of *Artemisia Capillaris* Extract in Rats, Japanese Journal of Pharmacology. (1981) 31, no. 5, 835–838, 10.1016/S0021-5198(19)52807-4. 7311175

[14] Jang S. I. , Kim Y.-J. , Lee W.-Y. , Kwak K. C. , Baek S. H. , Kwak G. B. , Yun Y.-G. , Kwon T.-O. , Chung H. T. , and Chai K.-Y. , Scoparone From *Artemisia capillaris* Inhibits the Release of Inflammatory Mediators in RAW 264.7 Cells Upon Stimulation Cells by Interferon-y Plus LPS, Archives of Pharmacal Research. (2005) 28, no. 2, 203–208, 10.1007/BF02977716, 2-s2.0-21844475945. 15789752

[15] Yan H. , Jung K. H. , Kim J. , Rumman M. , Oh M. S. , and Hong S.-S. , *Artemisia capillaris* Extract AC68 Induces Apoptosis of Hepatocellular Carcinoma by Blocking the PI3K/AKT Pathway, Biomedicine & Pharmacotherapy. (2018) 98, 134–141, 10.1016/j.biopha.2017.12.043, 2-s2.0-85038007033. 29253760

[16] WHO, FAO, & UNU , Protein and Amino Acid Requirements in Human Nutrition: Report of a Joint WHO/FAO/UNU Expert Consultation, 2027, WHO, 9–16.

[17] Wei W.-L. , Wang W.-J. , Chen H. , Lin S.-Y. , Luo Q.-S. , Li J.-M. , Yan J. , and Chen L.-L. , A Promising *Artemisia capillaris* Thunb. Leaf Proteins With High Nutrition, Applicable Function and Excellent Antioxidant Activity, Food Chemistry: X. (2024) 21, 101153, 10.1016/j.fochx.2024.101153. 38317669 PMC10838694

[18] Zhu S. , Du C. , Yu T. , Cong X. , Liu Y. , Chen S. , and Li Y. , Antioxidant Activity of Selenium-Enriched Peptides From the Protein Hydrolysate of *Cardamine violifolia* , Journal of Food Science. (2019) 84, no. 12, 3504–3511, 10.1111/1750-3841.14843, 31665556. 31665556

[19] Wang C. , Li B. , Wang B. , and Xie N. , Degradation and Antioxidant Activities of Peptides and Zinc–Peptide Complexes During In Vitro Gastrointestinal Digestion, Food Chemistry. (2015) 173, 733–740, 10.1016/j.foodchem.2014.10.066, 2-s2.0-84908538472, 25466083. 25466083

[20] Brodkorb A. , Egger L. , Alminger M. , Alvito P. , Assunção R. , Ballance S. , Bohn T. , Bourlieu-Lacanal C. , Boutrou R. , Carrière F. , Clemente A. , Corredig M. , Dupont D. , Dufour C. , Edwards C. , Golding M. , Karakaya S. , Kirkhus B. , le Feunteun S. , Lesmes U. , Macierzanka A. , Mackie A. R. , Martins C. , Marze S. , McClements D. J. , Ménard O. , Minekus M. , Portmann R. , Santos C. N. , Souchon I. , Singh R. P. , Vegarud G. E. , Wickham M. S. J. , Weitschies W. , and Recio I. , INFOGEST Static In Vitro Simulation of Gastrointestinal Food Digestion, Nature Protocols. (2019) 14, no. 4, 991–1014, 10.1038/s41596-018-0119-1, 2-s2.0-85063625000. 30886367

[21] Ke H. , Ma R. , Liu X. , Xie Y. , and Chen J. , Highly Effective Peptide-Calcium Chelate Prepared From Aquatic Products Processing Wastes: Stickwater and Oyster Shells, LWT. (2022) 168, 113947, 10.1016/j.lwt.2022.113947.

[22] Cui P. , Sun N. , Jiang P. , Wang D. , and Lin S. , Optimised Condition for Preparing Sea Cucumber Ovum Hydrolysate–Calcium Complex and its Structural Analysis, International Journal of Food Science and Technology. (2017) 52, no. 8, 1914–1922, 10.1111/ijfs.13468, 2-s2.0-85019270548.

[23] Liang R. , Li X. , Lin S. , and Wang J. , Effects on Functional Groups and Zeta Potential of SAP _1 < MW < 3kDa_ treated by pulsed electric field technology: Effects of PEF on SAP1 < MW < 3kDa, Journal of the Science of Food and Agriculture. (2017) 97, no. 2, 578–586, 10.1002/jsfa.7768, 2-s2.0-84973358563, 27098170. 27098170

[24] Zhang Y. , Ding X. , and Li M. , Preparation, Characterization and *In Vitro* Stability of Iron-Chelating Peptides From Mung Beans, Food Chemistry. (2021) 349, 129101, 10.1016/j.foodchem.2021.129101, 33540219. 33540219

[25] Jasim S. B. , Li Z. , Guest E. E. , and Hirst J. D. , Dichrocalc: Improvements in Computing Protein Circular Dichroism Spectroscopy in the Near-Ultraviolet, Journal of Molecular Biology. (2018) 430, no. 15, 2196–2202, 10.1016/j.jmb.2017.12.009, 2-s2.0-85039555085, 29258819. 29258819

[26] Micsonai A. , Wien F. , Kernya L. , Lee Y.-H. , Goto Y. , Réfrégiers M. , and Kardos J. , Accurate Secondary Structure Prediction and Fold Recognition for Circular Dichroism Spectroscopy, Proceedings of the National Academy of Sciences. (2015) 112, no. 24, 10.1073/pnas.1500851112, 2-s2.0-84935843728. PMC4475991 26038575

[27] Qin X.-Y. , Zhang J.-T. , Li G.-M. , Cai M.-Y. , Lu J. , Gu R.-Z. , and Liu W.-Y. , Selenium-Chelating Corn Oligopeptide as a Potential Antioxidant Supplement: Investigation of the Protein Conformational Changes and Identification of the Antioxidant Fragment Composition, International Journal of Food Engineering. (2020) 16, no. 4, 20190166, 10.1515/ijfe-2019-0166.

[28] Wang Y. , Bai H. , Wang S. , Wang R. , and Wang Z. , Casein Phosphopeptide-Calcium Chelate: Preparation, Calcium Holding Capacity and Simulated Digestion In Vitro, Food Chemistry. (2023) 401, 134218, 10.1016/j.foodchem.2022.134218, 36115235. 36115235

[29] Zhao L. , Huang Q. , Huang S. , Lin J. , Wang S. , Huang Y. , Hong J. , and Rao P. , Novel Peptide With a Specific Calcium-Binding Capacity From Whey Protein Hydrolysate and the Possible Chelating Mode, Journal of Agricultural and Food Chemistry. (2014) 62, no. 42, 10274–10282, 10.1021/jf502412f, 2-s2.0-84908426237, 25265391. 25265391

[30] Zhang C. , Du B. , Song Z. , Deng G. , Shi Y. , Li T. , and Huang Y. , Antioxidant Activity Analysis of Collagen Peptide-Magnesium Chelate, Polymer Testing. (2023) 117, 107822, 10.1016/j.polymertesting.2022.107822.

[31] Lobo V. , Patil A. , Phatak A. , and Chandra N. , Free Radicals, Antioxidants and Functional Foods: Impact on Human Health, Pharmacognosy Reviews. (2010) 4, no. 8, 10.4103/0973-7847.70902, 2-s2.0-78651391894. PMC3249911 22228951

[32] Fan C. , Wang X. , Song X. , Sun R. , Liu R. , Sui W. , Jin Y. , Wu T. , and Zhang M. , Identification of a Novel Walnut Iron Chelating Peptide With Potential High Antioxidant Activity and Analysis of its Possible Binding Sites, Food. (2023) 12, no. 1, 10.3390/foods12010226. PMC9818316 36613440

[33] He Y. , Yang P. , Ding Y. , Chen M. , Guo R. , Duan Y. , Zhang H. , and Ma H. , The Preparation, Antioxidant Activity Evaluation, and Iron-Deficient Anemic Improvement of Oat (*Avena sativa* L.) Peptides-Ferrous Chelate, Frontiers in Nutrition. (2021) 8, 687133, 10.3389/fnut.2021.687133, 34235170. 34235170 PMC8256796

[34] Sun R. , Liu X. , Yu Y. , Miao J. , Leng K. , and Gao H. , Preparation Process Optimization, Structural Characterization and In Vitro Digestion Stability Analysis of Antarctic Krill (*Euphausia superba*) Peptides-Zinc Chelate, Food Chemistry. (2021) 340, 128056, 10.1016/j.foodchem.2020.128056, 33032152. 33032152

[35] Hou Y. , Chen X. , Zhang M. , Yang S. , Liao A. , Pan L. , Wang Z. , Shen X. , Yuan X. , and Huang J. , Selenium-Chelating Peptide Derived From Wheat Gluten: In Vitro Functional Properties, Foods. (2024) 13, no. 12, 10.3390/foods13121819, 38928761. PMC11203129 38928761

[36] Fan J. , Schiemer T. , Vaska A. , Jahed V. , and Klavins K. , Cell Via Cell Viability Assay Changes Cellular Metabolic Characteristics by Intervening With Glycolysis and Pentose Phosphate Pathway, Chemical Research in Toxicology. (2024) 37, no. 2, 208–211, 10.1021/acs.chemrestox.3c00339. 38191130 PMC10880084

